# Prof. Trousseau

**Published:** 1854-11

**Authors:** 


Prof. Trousseau recommends the extract of belladonna, in doses of one-fifth of a grain, three times a day, as a remedy par excellence for habitual constipation. With great deference to this distinguished savan of France, we would suggest that his remedy will be far more successful if he will add thereto 3 grs. of P. Rhei and 1 gr. of P. Ipecac; these being the constituents of Prof. Chapman's "Peristaltic Persuaders." The latter will succeed when the belladonna fails.—*N. Y. Med Gaz.*

